# Characterization and cytotoxic effect of biogenic silver nanoparticles on mitotic chromosomes of *Drimia polyantha* (Blatt. & McCann) Stearn

**DOI:** 10.1016/j.toxrep.2018.08.018

**Published:** 2018-08-31

**Authors:** Azharuddin Daphedar, Tarikere C. Taranath

**Affiliations:** Environmental Biology Laboratory, P. G. Department of Studies in Botany, Karnatak University, Dharwad 580003, Karnataka, India

**Keywords:** Green synthesis, Silver nanoparticles, Characterization, Mitotic index, Chromosomal aberrations

## Abstract

Noble metal nanoparticles afford a tool for investigation and its application in biological systems has had the greatest impact in biology and biomedicine. The present work reports an ecofriendly approach for the synthesis of silver nanoparticles (AgNPs) using an aqueous leaf extract of *Getonia floribunda.* The silver nanoparticles were characterized by using following instruments viz. UV–vis spectrophotometer, FTIR, XRD AFM and HR-TEM. The UV–vis spectrum showed a characteristic absorption peak at 404 nm. FTIR data reveals the possible involvement of various functional groups for reduction and biocapping of AgNPs. XRD data confirmed the crystalline nature of silver nanoparticles. Morphology, size and distribution of the AgNPs were determined by using AFM and HR-TEM. The average size of AgNPs ranges between 10 and 25 nm and are spherical in shape. The silver nanoparticles were evaluated for their cytotoxic effect on mitotic chromosomes of root meristematic cells of *D. polyantha* using different concentrations viz. 4, 8, 12 and 16 μg/ml at the time interval of 6, 12, 18 and 24 h. It is evident from the results that the higher concentration of AgNPs found to inhibit mitotic index and caused chromosomal abnormalities such as chromosomal bridge, sticky chromosomes, laggard anaphase, diagonal anaphase, c-metaphase and chromosomal breaks. Therefore, it can be concluded that higher concentrations of silver nanoparticles may induce significant inhibition of root meristem activity and causing DNA damage.

## Highlights

• Synthesis of AgNPs by leaf extract of *G. floribunda*.
• Inhibition of mitotic index (MI) at higher concentration of AgNPs.
• Cytotoxic effect is directly proportional to the concentration of AgNPs.
• Higher concentrations of silver nanoparticles induce significant inhibition of root meristem activity and DNA damage causing cell death.

## 1 Introduction

In the present scenario, the field of nanoscience is rapidly growing day by day in various areas of research, having a wide range of applications in the field of medicine, biology, material science, physics and chemistry [1,2]. Bioinspired nanoparticles, structurally ranging from approximately 10–100 nm in size, have received enormous attention over the physical and chemical synthesis of nanoparticles because their production is simple, cost effective, energy efficient, ecofriendly and do not produce hazardous chemicals during the process [3]. Currently, biosynthesis of metal nanoparticles by bacteria, fungi, microbes arthropods, agro- wastes, enzyme plant derived pigments and green plants [[4], [5], [6]] have been well documented. Silver is a fairly exceptional metal that naturally occurs on the surface of the earth. Earlier, it has been used before the advent of antibiotic drug in medical devices [7]. Recently, silver nanoparticles and their applications are being widely used in the field of nanomedicine to treat atrocious diseases like cancer, AIDS, diabetes, malaria, tuberculosis, antioxidant, anticoagulants and thrombolytic agents [[8], [9], [10], [11], [12], [13], [14], [15], [16], [17], [18]]. Therapeutically, cancer is one of the major health problems all over the world which can cause toxicity and increased risk of oxidative DNA damage leading to cell death.

Researchers have demonstrated that the silver nanoparticles induce strong cytotoxicity in broad spectrum of cells including germ line stem cells, messenchymal stem cells (hMSCs), BRL 3 A rat liver cells, NIH3T3 cells, HepG2 human hepatoma cells, normal human lung fibroblasts (IMR-90), human glioblastoma cells (U251), human normal bronchial epithelial (BEAS-2B) cells and HeLa cells [[19], [20], [21], [22], [23], [24], [25], [26], [27], [28], [29]]. On the contrary, there are few reports on silver and zinc nanoparticle toxicity on root tip mitotic cells of *Allium cepa, Vicia faba, Allium sativum* and *Drimia indica* [30,31]. These plant systems have been frequently used to study cytotoxic and genotoxic effects of nanoparticles on mitotic cell division and they have been considered as well model genetic system [32]. Yekeen et al. [33] and Yekeen et al. [34] have been reported that the cytotoxic effect of AgNPs on root meristems of *Allium cepa* used as a model of biological system. Hence, the present study was undertaken to investigate cytotoxic effect of silver nanoparticles on mitotic chromosomes of meristimetic root tip cells of *D. polyantha*.

## 2 Materials and methods

**Fig. 1:**
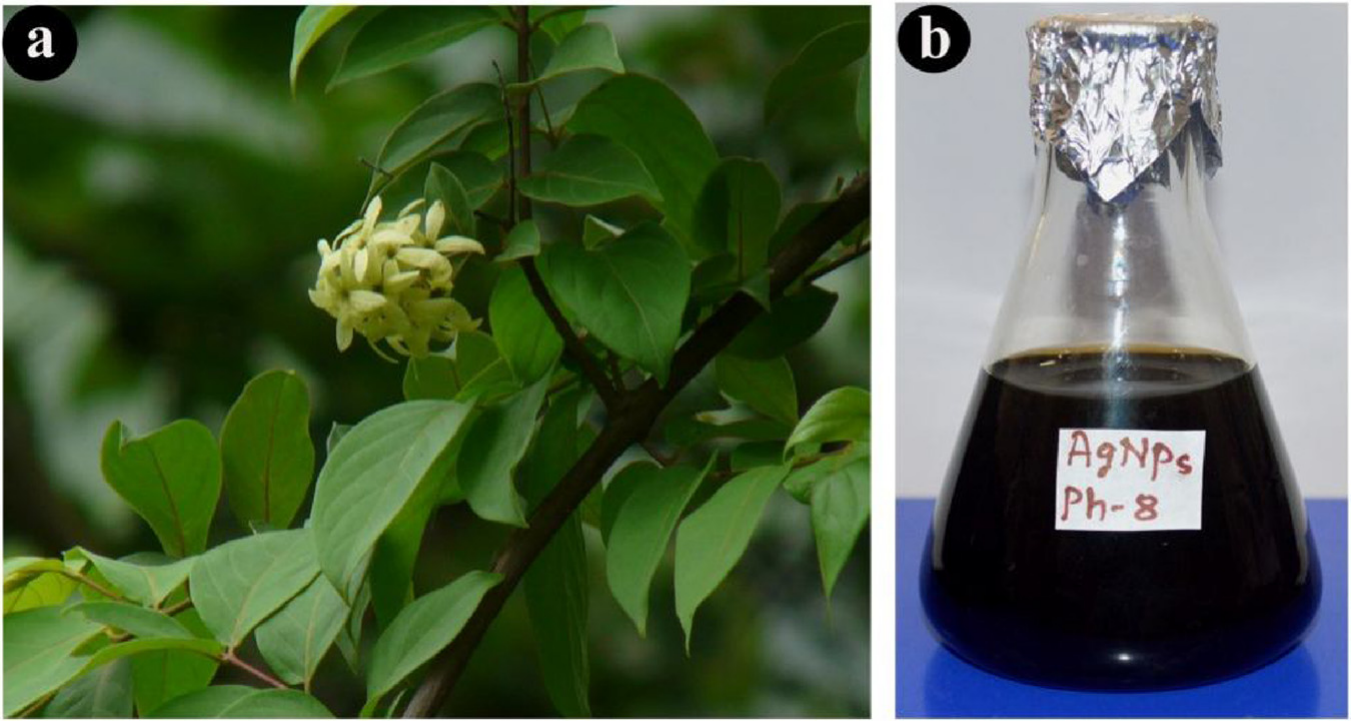
a) Leaf of *Getonia floribunda* b) Formation of silver nanoparticles (AgNPs).

The fresh leaves of *Getonia floribunda* Roxb. (Synonym (*Calycopteris floribunda* (Roxb.) Lam. ex Poir.)) belonging to the family Combretaceae (Fig. 1a) were collected from Botanical garden of Karnatak University campus Dharwad, Karnataka, India.

### 2.1 Preparation of leaf extract

The collected leaves were initially rinsed with running tap water and then washed with double distilled sterile water to remove adhering dust and impurities. The leaves were dried under the shade and incised into small pieces with scissor. Then, 10 g leaves were weighed and boiled for 15–20 minutes in 100 mL Milli Q water and extract was filtered through Whatman filter paper No. 1. The extract was stored in refrigerator for further analysis.

### 2.2 Synthesis of silver nanoparticles

About 5 ml of leaf extract was added to 95 ml of 1 mM silver nitrate (AR) solution in a 250 ml of Erlenmeyer flask. After, 15–20 min, the color of reaction mixture changed from yellow to dark brown (Fig. 1b), indicating the formation of silver nanoparticles and reduction of Ag + ions.

### 2.3 Characterization of silver nanoparticles

The synthesis of silver nanoparticles were determined by UV–vis spectrophotometery (Jasco Corporation, Tokyo, Japan) by recording the spectra between 300–600 nm at the resolution of 1 nm. Further, the reaction mixture was centrifuged at 3500 rpm (Remi R-8C) for 45 min. The process of centrifugation and re-suspension was repeated for 2–3 times. The purified suspension was dried at 60 °C in an oven to obtain the powder form and analyzed further by Fourier Transform Infrared spectroscopy (FTIR). The dried powder of leaf sample was mixed with KBr to obtain KBr pellets and spectrum was obtained at the range of 4000–400 cm^−1^ to identify the possible biomolecules involved in the formation silver nanoparticles. X-ray diffractometer (XRD) was used to examine particle size and crystalline nature of the silver nanoparticles. The AFM samples were prepared by spin coating the AgNPs solution into the glass slide. The prepared slides were air-dried at 28 °C in room for 12 h and subjected to AFM analysis. The particle size, shape and surface morphology of silver nanoparticles were analyzed using an atomic force microscopy (AFM) and high resolution transmission electron microscopy (HR-TEM).

### 2.4 Experimental design

Fresh, healthy and disease free bulbs of *D. polyantha* (2n = 20) were collected from Shivaji University campus, Kolhapur (Maharashtra). The outer dry scales were removed from bulbs and the bottom roots were scraped without destroying root primordia. The bulbs were allowed to grow in glass coupling jar containing sterile distilled water. When the newly emerged roots grew 2 to 3 cm in length, they were treated with different concentration of silver nanoparticles suspension viz. 4, 8, 12 and 16 μg/ml for 24 h at the interval of 6, 12, 18 and 24 h. After treatment, root tips were excised and washed with distilled water and treated root tips were fixed in Carnoy’s fluid (ethyl alcohol; glacial acetic acid 3:1) for 24 h, transferred to 70% ethyl alcohol and stored in refrigerator at 10–15 °C for cytological studies.

### 2.5 Squash preparation

Root squash was prepared by following the literature method of Sharma and Sharma [35]. The root tips were hydrolyzed in 1 N HCl at 60 °C for 1–2 min and stained with 2% aceto orcein for 10 min. Each treatment was performed in triplicates and a minimum of 600 cells was counted in each slide, for both control and treated root tips. The effect of silver nanoparticles on mitotic index (MI) and chromosomal abnormalities (CAs) was determined using the following formula:

$$MI\left(\%\right)=\frac{Number\;of\;cells\;in\;mitosis}{Total\;number\;of\;cells}\times \;100$$

$$CA\;\left(\%\right)=\frac{Total\;number\;of\;chromosomal\;abnormality}{Total\;number\;of\;dividing\;cells}\times \;100$$

### 2.6 Stastical analysis

Statistical analysis of data was carried out by using SPSS windows software version 20 followed by two-way ANOVA and Tukey test.

## 3 Results and discussion

### 3.1 Characterization of nanoparticle

#### 3.1.1 UV–vis spectroscopic analysis

**Fig. 2:**
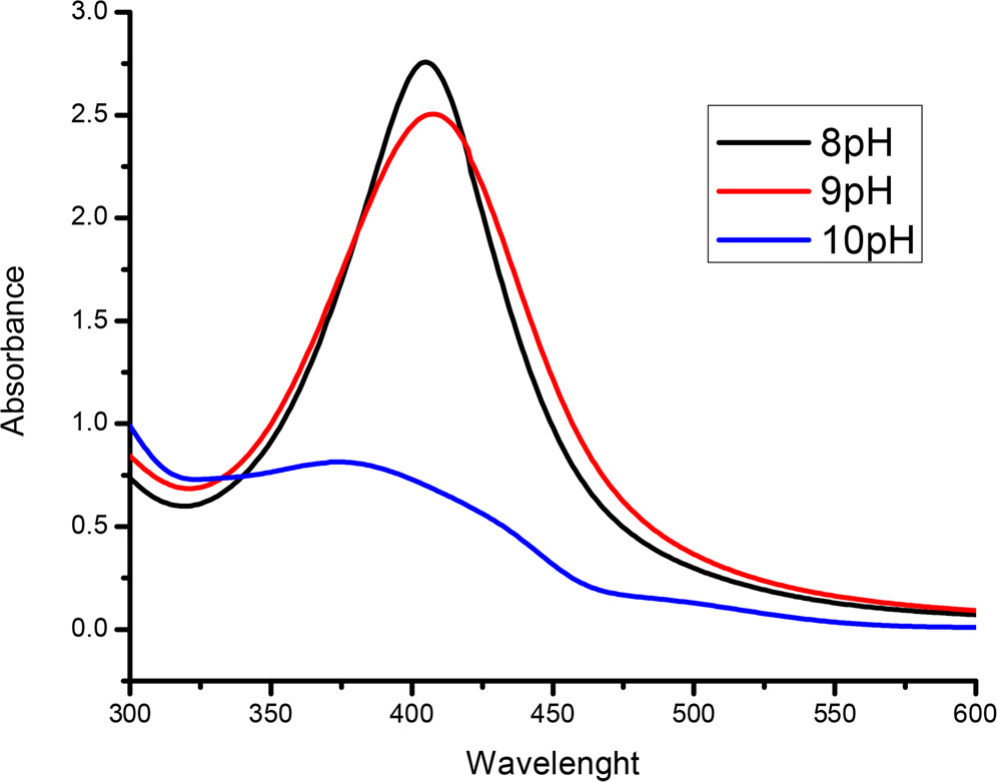
UV–vis absorption spectra of AgNPs synthesized by leaf extract of *G.* floribunda.

The color of reaction mixture changed from light yellow to dark brown indicating the formation of silver nanoparticles and this was further confirmed by using UV–vis spectrophotometry (Fig. 1b). The reduction of silver ions was due to the excitation of surface plasmon resonance (SPR) of the AgNPs [[36], [37], [38], [39]]. The spectrum of stabilized silver nanoparticles showed the broader absorption peak at 408 nm and 412 nm in red shift indicating the formation of silver nanoparticles with larger size at pH 9 and pH 10 respectively. The blue shift indicates the formation of small sized AgNPs at 404 nm absorption peak at pH 8. (Fig. 2).

#### 3.1.2 Fourier transmission infrared spectroscopic analysis

**Fig. 3:**
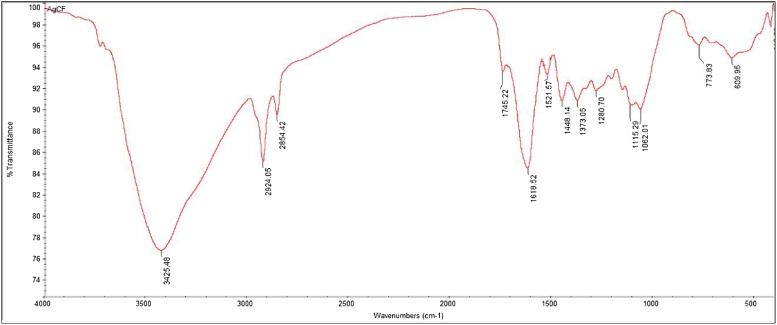
Fourier transform infrared spectroscopy of AgNPs synthesized by aqueous leaf extract of *G. floribunda*.

FTIR measurements were carried out to identify the biomolecules in the aqueous leaf extract of *G. floribunda*. The bioreduction and biocapping of silver ions leading to the formation of silver nanoparticles (Fig. 3 and Table 1) shows the absorption peaks at 3425, 2924, 1745, 1618, 1448, 1280, 1062 and 773 cm^−1^ respectively. The peak at 3425 cm^−1^ may be due to the O—H stretching of alcohols and phenols, the band at 2924 cm^−1^ is attributed to C—H stretching of carboxylic acids and methylene associated with proteins [3]. The band at 1745 cm^−1^corresponds to C=O stretching region of COOH or ester groups [40]. The band 1618 cm^−1^ could also belong to asymmetric and symmetric stretching of the carboxylate anion group (COO) or aromatic alkene C=C group. The band of 1448 cm^−1^ was due to C—C stretching of aromatic amines. The band of 1280 cm^−1^ corresponds due to C—N stretching of aromatic amines and 1062 cm^−1^ due to the C—N stretching of aliphatic amines. The band of 773 cm^−1^ appears generally due to C—Cl stretching of alkyl halides. The FTIR data reveals that the phytochemicals in *G. floribunda* leaf extract were responsible for the reduction, capping and stabilization of silver nanoparticles.

**Table 1:** Showing FTIR absorption bands and their associated functional groups involved in the biofabrication of silver nanoparticles.

Serial No.	Absorption band (cm^−1^)	Functional groups
1	3425	Alcohols and phenols
2	2924	Carboxylic acids and methylene
3	1745	Carbonyl groups
4	1618	Asymmetric and symmetric stretching of the carboxylate anion group (COO) or aromatic alkene C=C group.
5	1448	Aromatics
6	1280	Aromatic amines
7	1062	Aliphatic amines
8	773	Alkyl halides

#### 3.1.3 X-ray diffraction analysis

**Fig. 4:**
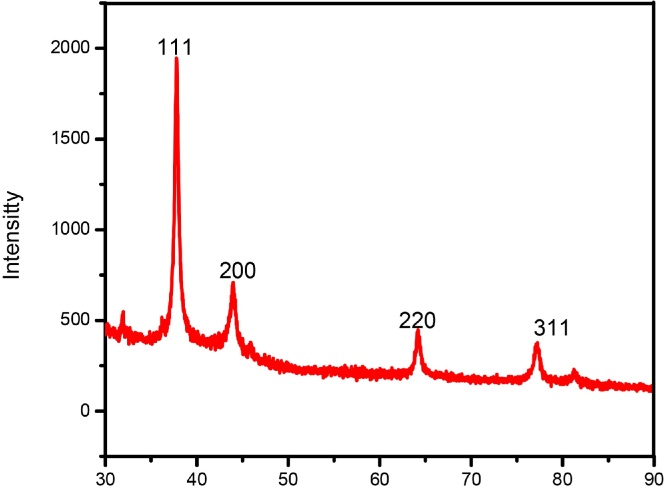
X-ray diffraction spectrum of biogenic AgNPs using leaf extract of *G. floribunda.*

Crystalline nature of biogenic silver nanoparticles was analyzed by using X-Ray diffractometer (Fig. 4). This method was used to identify the phase composition, crystalline structure, orientation and size of the synthesized silver nanoparticles. The XRD intense peaks detected in the 2θ angles at (111), (200), (220) and (311) orientations. The intense peak (111) relatively higher than the usual values, which indicate the nano size of silver particles. Further, it was confirmed by using Scherrer’s formula $D=\frac{Kʎ}{\beta \;cos\theta }$ [[41], [42], [43]]. The average size of the nanoparticles was found to be approximately14.6 nm.

#### 3.1.4 Atomic force microscopy (AFM) and high resolution transmission electron microscopy (HR-TEM) analysis

**Fig. 5:**
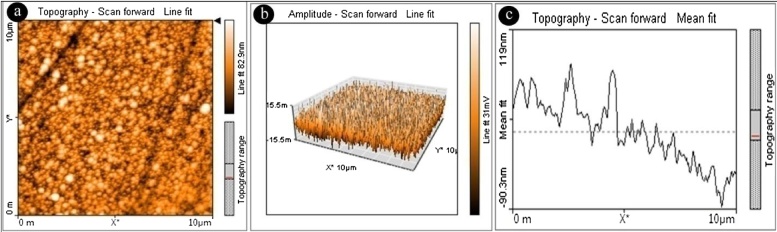
Atomic force microscopy images of biogenic AgNPs using leaf extract of *G. floribunda* a) 2D image b) 3D image of AgNPs, and c) Distribution of particle size of AgNPs.

**Fig. 6:**
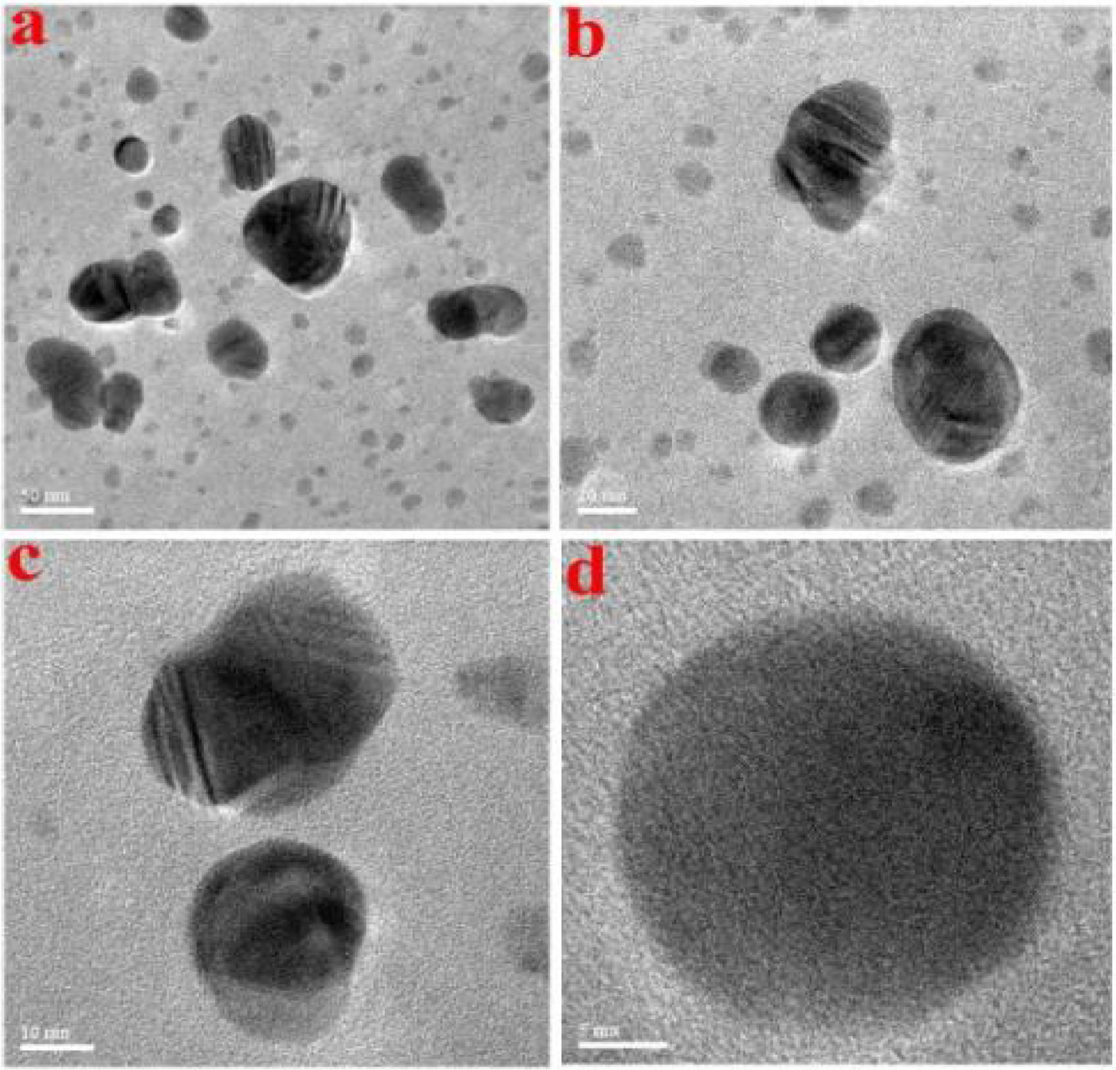
HR-TEM images of synthesized AgNPs using leaf extract of *G. floribunda*.

Size and shape of silver nanoparticles were determined by using Atomic Force Microscopy (AFM). Fig. 5(a) represents two dimensional image of silver nanoparticles and are monodispersed and spherical in shape with an average size range between 10 and 25 nm. The Fig. 5(b and c) represents three dimensional and topographical images of silver nanoparticles. HR-TEM imaging (Fig. 6) confirmed the size and shape of the silver nanoparticles. The silver nanoparticles are crystalline and spherical in shape showing lattice fringes with an average size ranging between 10 and 22 nm.

#### 3.1.5 Cytotoxic and genotoxic assay

**Fig. 7:**
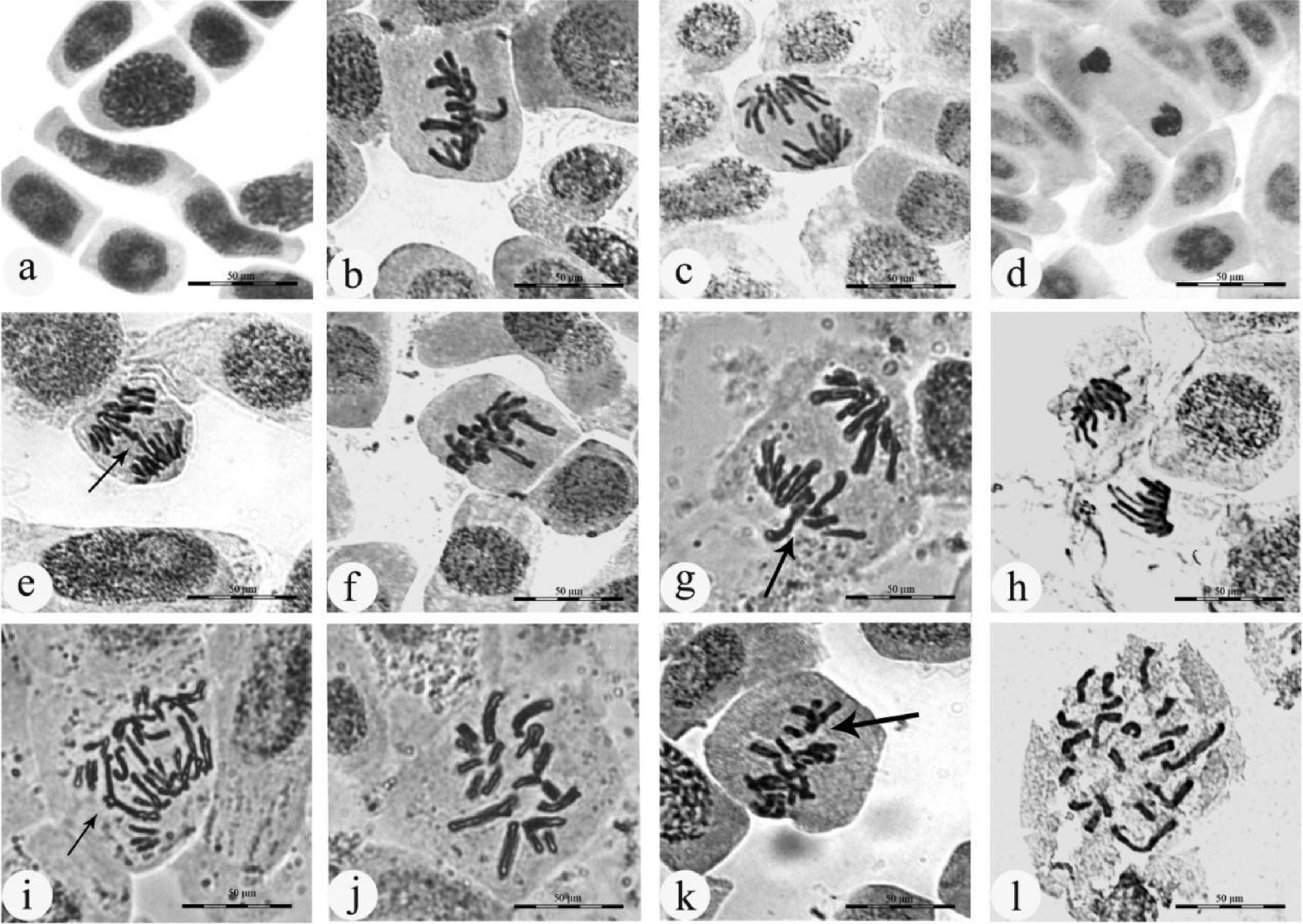
Chromosomal abberations of root meristimetic cells of *D. polyantha* treated with different concentrations of AgNPs solution and control (without AgNPs) a) Normal prophase b) Normal metaphase c) Normal anaphase d) Normal telophase e) Anaphase bridge f) Sticky metaphase g) Laggard anaphase h) Diagonal anaphase i) Disturbed anaphase j) c-metaphase k) Disturbed metaphase l) Distribution of chromosomes.

The present investigation revealed that the mitotic index was significantly decreased with increased concentration of silver nanoparticles in a dose dependent manner (Fig. 7, Fig. 9 and Table 2). The mitotic index was highest in control with a value of (70.08 ± 2.82) and lowest in 16 μg/ml concentration of AgNPs solution (37.90 ± 2.44) at 24 h. It is evident from the observations that the MI was significantly decreased at each exposure time, as compared to control. Few reports suggest that decrease in the MI was due to the influence of nanosilver, resulting from the effect of test agent on the growth and development of exposed organisms [44]. The results were found to be statistically significant at *p* < 0.05. The proportion of dividing cells decreased in the prophase stage at 24 h treatment period at 16 μg/ml concentration of AgNPs solution, while in metaphase, anaphase and telophase, it is dwindled in almost all analyzed cells when compared to control. Similar results have been reported with treatment of chromium (III) oxide nanoparticles on *Allium cepa* [45]. Our previous study [46] reported that possible detrimental cytotoxic effect on mitotic chromosomes in *D. indica*, induced percentage of genotoxicity is directly proportional to the concentration of biogenic AgNPs. The mitotic activity was decreased due to interference of AgNPs with normal mitotic cell cycle. A slower progression of cells to S phase (DNA synthesis) and blockage of G2 phase, which under very severe toxicant treatment normally leads to cell death [47,48], might occur also in the plant system. Silver nanoparticles destroy cell permeability of bacterial outer membrane; hinder respiration, growth of cells and structure of membrane, leading to cell disintegration and death ultimately [49, 50]. It has been proved that silver nanoparticles exhibit antimicrobial activity because of the interaction of silver nanoparticles with sulphur and phosphorous containing biomolecules in bacterial cell, when these nanoparticles internalized by the bacterial cell, initiate cell death through the attack of the respiratory chain and cell division [51]. In addition, gold nanoparticles can inhibit pathogens by damaging the cell wall and also by generating leakage of cytoplasmic ingredients including acidification of intracellular environment [15]. Also, silver nanoparticles, entering into cells via cell wall, may indeed cause DNA damage [52]. To date, a variety of plant compounds have been studied extensively such as antimutagenic, anticarcinogenic, and antigenotoxic activities [53]. However, AgNPs, Al_2_O_3_ and ZnO nanoparticles have shown dose-dependent inhibition of mitotic index [30, 31, 54].

**Table 2:** Effect of AgNPs on mitotic index (MI) and chromosomal aberrations (CAs) of *D. polyantha* root tip cells.

Duration (h)	Treatment of AgNP (μg/ml)	Total No. of cells examined	No. of dividing cells	No. of non dividing cells	P (%)	M (%)	A (%)	T (%)	MI (%)	CA (%)
	Control	2432	1705	727	91.61 ± 3.10	3.91 ± 1.32	2.78 ± 1.13	1.66 ± 0.66	70.08 ± 2.82	0 ± 0
6	4	2172	1408	764	84.87 ± 1.34	3.89 ± 0.99	2.66 ± 1.31	0.99 ± 0.17	65.02 ± 1.64	6.95 ± 0.24
	8	2151	1323	828	87.89 ± 1.63	2.99 ± 0.51	2.12 ± 0.49	1.21 ± 0.21	61.44 ± 1.89	5.38 ± 0.95
	12	2182	1220	962	87.67 ± 2.77	3.22 ± 0.90	2.32 ± 0.80	1.51 ± 0.86	56.00 ± 5.99	5.02 ± 0.74
	16	2106	1056	1050	83.44 ± 2.61	3.97 ± 0.18	2.87 ± 1.25	1.15 ± 0.80	50.38 ± 5.85	8.26 ± 2.62
12	4	2178	1404	774	88.66 ± 4.26	3.03 ± 1.09	1.84 ± 0.09	0.85 ± 0.39	64.50 ± 1.45	5.50 ± 3.20
	8	2014	1225	789	84.33 ± 3.76	4.20 ± 0.71	1.99 ± 0.66	1.45 ± 0.63	60.77 ± 1.32	7.99 ± 3.36
	12	2045	1196	849	85.21 ± 3.54	3.40 ± 0.55	1.82 ± 0.48	1.83 ± 0.37	58.57 ± 1.84	7.54 ± 2.50
	16	2056	1134	922	82.54 ± 1.86	3.55 ± 0.66	2.28 ± 0.97	1.49 ± 0.25	55.02 ± 2.63	10.42 ± 1.85
18	4	2082	1289	793	88.75 ± 2.22	3.16 ± 0.81	2.00 ± 0.39	0.84 ± 0.10	61.94 ± 2.20	5.21 ± 1.43
	8	1984	1209	775	87.64 ± 3.14	3.56 ± 0.68	2.24 ± 0.95	0.80 ± 0.31	60.88 ± 1.86	6.11 ± 1.25
	12	2139	1164	975	86.97 ± 3.00	3.22 ± 1.62	1.97 ± 0.38	0.77 ± 0.53	57.09 ± 1.08	1.02 ± 1.54
	16	1980	1024	956	85.23 ± 1.52	3.12 ± 0.65	1.94 ± 0.42	1.17 ± 0.58	51.73 ± 1.08	8.20 ± 1.27
24	4	2097	1335	762	86.70 ± 3.03	3.39 ± 0.81	2.03 ± 0.76	0.83 ± 0.52	63.58 ± 2.78	14.79 ± 12.27
	8	2143	1301	842	86.65 ± 0.70	3.91 ± 0.69	1.77 ± 0.17	0.83 ± 0.10	60.67 ± 3.45	7.88 ± 1.34
	12	2019	1144	875	80.68 ± 3.30	4.20 ± 0.77	2.51 ± 0.71	1.03 ± 0.42	52.26 ± 8.70	11.53 ± 3.25
	16	2076	789	1287	68.37 ± 2.06	6.90 ± 2.16	2.70 ± 0.96	1.89 ± 0.29	37.90 ± 2.44	Cell damage

SD- Standard Deviation.

Significant at 5% level (*p* < 0.05).

*Note:* MI- Mitotic index, CA- Chromosomal abnormalities.

Chromosomal aberrations (CAs) were observed in almost all root tip meristematic cells of *D. polyantha* treated with different concentrations of silver nanoparticles (Figs. 7, 9 and Table 2). Nanoparticles are responsible for change in chromosomal behavior either structurally or numerically, resulting in the breakage and reunion of chromosomal material [[55], [56], [57]]. Silver nanoparticles are found to affect the viscosity of cytoplasm leading to abnormal spindle behavior causing chromosomal abnormalities. The CAs was increased with increasing concentration of silver nanoparticle up to 12 μg/ml and complete cell death was observed in 16 μg/ml concentration at 24 h duration. Results are statistically significant (*p* < 0.05), as compared to control. The maximum proportion of chromosomal aberrations was recorded in 4 μg/ml of AgNPs suspension (14.79±12.27%) for 24 h duration. The present investigation shows that the induction of CAs was due to the increasing concentration of AgNPs suspension. When these nanoparticles penetrate in to the root system, they can cause a number of mitotic abnormalities such as bridges, sticky, laggard, diagonal, c-metaphase, multipolar anaphase and disturbed metaphase.

The maximum number of chromosomal bridges was observed at 12 μg/ml concentration of AgNPs solution (2.30 ± 1.12) for 24 h duration Fig. 9. Bridges arose due to the irreversible effect of Ag^+^ ions on the dicentric chromosomes and these chromosomes unequally exchange their chromosomal fragments, undergoing translocation resulting into the formation of bridge called chromosomal bridge or anaphase bridge [58]. Maximum number of 3.60 ± 1.14 chromosomal stickness was recorded at 12 μg/ml concentration of AgNPs solution for 24 h duration. Chromosomal stickyness was due to excessive formation of nucleoproteins, condensation of chromosomes, DNA depolymerization and it may lead to toxic effect on cell and causes cell death or senescence [57]. Laggard chromosome was observed maximum in 12 h duration of treatment at 16 μg/ml concentrations (0.96 ± 0.25) of silver nanoparticles solution. The laggard chromosome was due to the interference of AgNPs in the cell and they may have effect on spindle fiber resulting in failure of acentric chromosomes. These chromosomal fragments move to the opposite pole and may cause delayed metaphase or prophase [58]. Further, the diagonal chromosomes (1.43 ± 0.44) were recorded at 16 μg/ml concentration of AgNPs solution for 12 h (Fig. 9) El-ghamery et al. [59] Suggesting that the two sets of anaphasic chromosomes do not lie in the same alignment because the silver ions significantly affect spindle fibers.

**Fig. 8:**
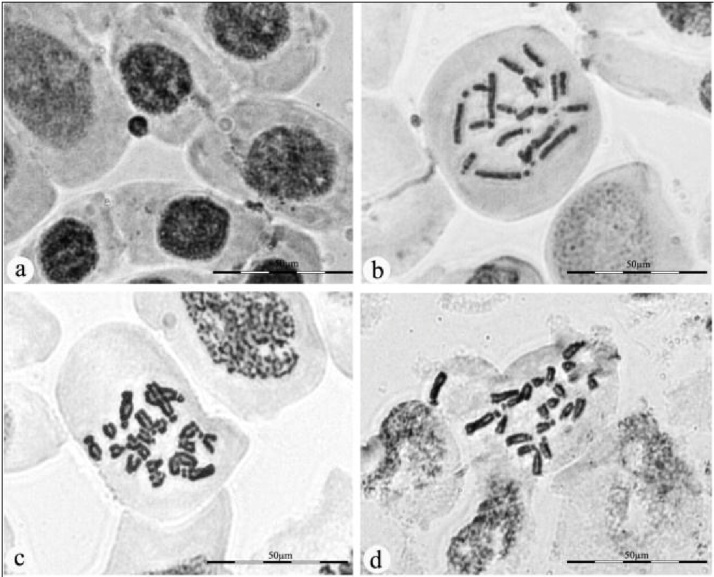
Chromosomal aberrations and chromosome damage in meristimetic root cells of *Drimia polyantha* a) micronucleus b) whole chromosomes set is normal c) four damaged chromosome d) one damaged chromosome.

The most common type of abnormality was observed in the stage of C shaped metaphase and disturbed metaphase. The maximum frequencies (2.68 ± 0.30) of C- metaphase were recorded at 8 μg/ml concentration of AgNPs for 24 h duration. C- metaphase causes inhibition of spindle formation, induce the risk of aneuploidy and sticky chromosomes toxic irreversible effect to the cell causing its death [60, 61]. Another type of mitotic abnormalities is disturbed metaphase; the frequency of disturbed chromosome was highest in 16 μg/ml concentration (1.43 ± 0.36) for 12 h duration of exposure. It was recorded mainly due to the disturbances of spindle apparatus and depolymerization of spindle fibers resulting in the shifting of poles during the stage of anaphase and metaphase [62]. The failure of spindle function, change in viscosity of cell sap leading to formation of micronucleus (Fig. 8a) for instance, chromosome breaks and losses. These findings suggest that the higher concentration of silver nanoparticles can induce toxicity and can affect the whole genome resulting into the DNA damage [63, 64]. Previous reports [30, 26, 31] on mammalian and plant cells have proved that AgNPs accumulate in cytoplasm and may lead to toxic effect on mitochondria and nucleus, causing uncoupling of respiration and increased oxidative stress which leads to DNA breakage, base modification and cross linking of DNA damage, which can be repaired with or without cell cycle arrest [65, 66]. The present study demonstrated that the frequency of chromosomal aberrations is inversely proportional to the mitotic index (MI) with increased concentrations of AgNPs in a dose dependent manner, which results in DNA breakage, inhibition of DNA synthesis and altered DNA replication [67]. The present investigation exhibit that AgNPs synthesized by *G. floribunda* leaf extract cause DNA damage, which leads to cell death at higher concentration of silver nanoparticle solution.

**Fig. 9:**
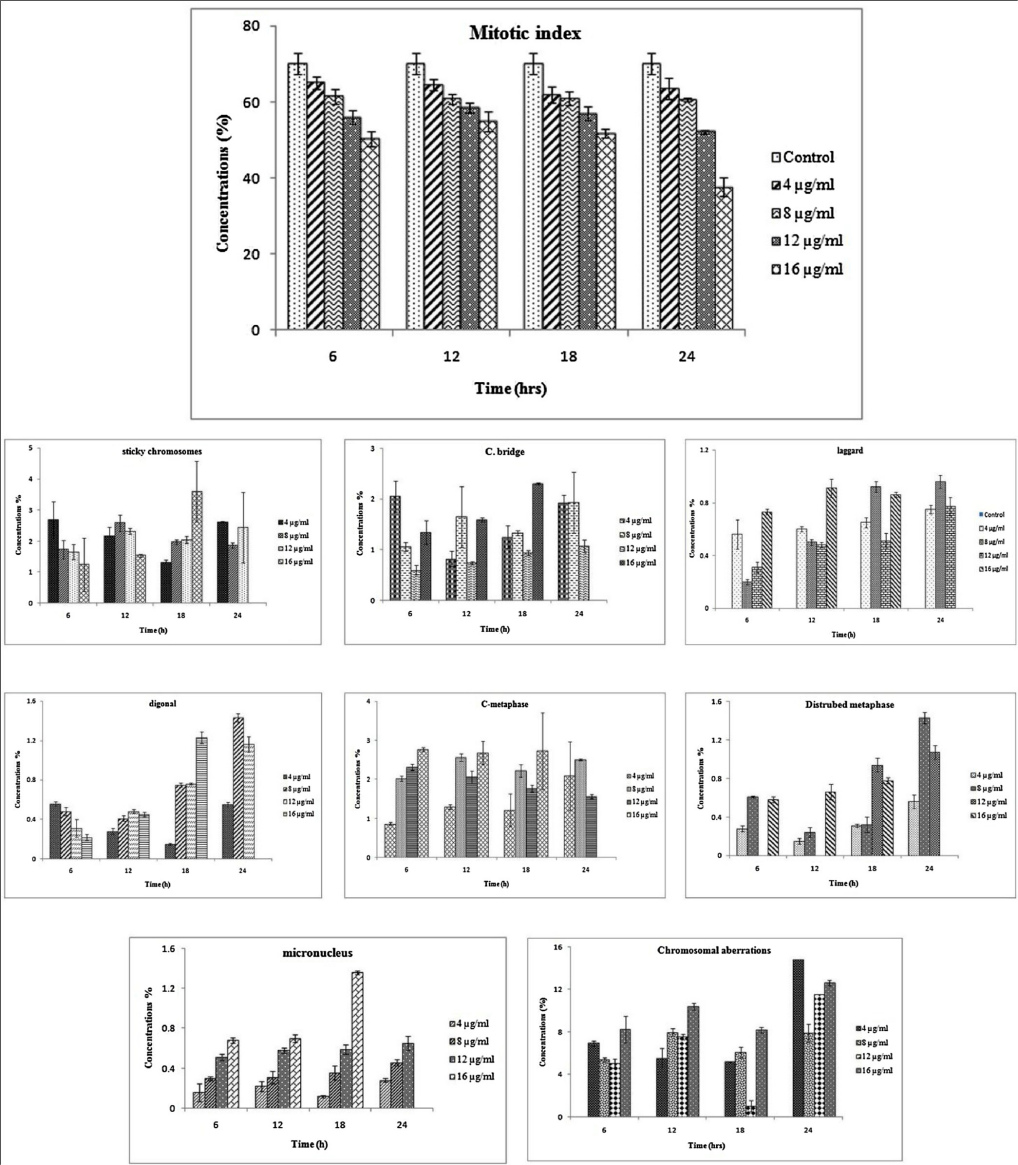
The mitotic index (MI) and Chromosomal aberrations (CAs) in root tip meristimetic cells of *D. polyantha*, scored at different exposure periods (6–24 h), at different concentrations (4–16 μg/ml), found to be stastically significant at (p < 0.05), when compared to the control.

## 4 Conclusion

The synthesis of silver nanoparticles by *G. floribunda* leaf extract has been demonstrated as a reliable and cost effective method. FTIR data revealed that biomolecules viz. proteins, carboxylic acids, flavonols, alcohols and phenols were involved in bioreduction and biocapping of silver nanoparticles. Crystalline nature of silver nanoparticles was confirmed by XRD. Biogenic silver nanoparticles were spherical in shape with an average size range from 10 to 22 nm. Silver nanoparticles induced cytotoxic effect on root tip meristimetic cells of *D. polyantha* and responsible for chromosomal abnormalities. The cytotoxic effect and chromosomal abnormalities were found to be dose and duration dependent. It is found that the higher concentrations of AgNPs inhibit the division of mitotic cell and increased chromosomal aberrations leading to cell death. This potential of AgNPs may be explored for cancer treatment.

## Conflict of interest

The authors declare that there is no conflict of interest.
